# Starting to smoke: a qualitative study of the experiences of Australian indigenous youth

**DOI:** 10.1186/1471-2458-12-963

**Published:** 2012-11-10

**Authors:** Vanessa Johnston, Darren W Westphal, Cyan Earnshaw, David P Thomas

**Affiliations:** 1 Menzies School of Health Research, Institute of Advanced Studies, Charles Darwin University, PO Box 41096, Casuarina, Northern Territory 0811 Australia; 2 Lowitja Institute, Charles Darwin University, PO Box U364, Casuarina, Northern Territory 0815 Australia

**Keywords:** Aboriginal, Youth, Smoking, Tobacco, Australia

## Abstract

**Background:**

Adult smoking has its roots in adolescence. If individuals do not initiate smoking during this period it is unlikely they ever will. In high income countries, smoking rates among Indigenous youth are disproportionately high. However, despite a wealth of literature in other populations, there is less evidence on the determinants of smoking initiation among Indigenous youth. The aim of this study was to explore the determinants of smoking among Australian Indigenous young people with a particular emphasis on the social and cultural processes that underlie tobacco use patterns among this group.

**Methods:**

This project was undertaken in northern Australia. We undertook group interviews with 65 participants and individual in-depth interviews with 11 youth aged 13–20 years led by trained youth ‘peer researchers.’ We also used visual methods (photo-elicitation) with individual interviewees to investigate the social context in which young people do or do not smoke. Included in the sample were a smaller number of non-Indigenous youth to explore any significant differences between ethnic groups in determinants of early smoking experiences. The theory of triadic influence, an ecological model of health behaviour, was used as an organising theory for analysis.

**Results:**

Family and peer influences play a central role in smoking uptake among Indigenous youth. Social influences to smoke are similar between Indigenous and non-Indigenous youth but are more pervasive (especially in the family domain) among Indigenous youth. While Indigenous youth report high levels of exposure to smoking role models and smoking socialisation practices among their family and social networks, this study provides some indication of a progressive denormalisation of smoking among some Indigenous youth.

**Conclusions:**

Future initiatives aimed at preventing smoking uptake in this population need to focus on changing social normative beliefs around smoking, both at a population level and within young peoples’ immediate social environment. Such interventions could be effectively delivered in both the school and family environments. Specifically, health practitioners in contact with Indigenous families should be promoting smoke free homes and other anti-smoking socialisation behaviours.

## Background

There are significant disparities in tobacco use among young people worldwide. In the United States, American Indian and Alaskan Native young adults have the highest prevalence of current cigarette smoking of all ethnic subgroups in the country [1]. In Australia, 42% of Indigenous Australians are current smokers by early adulthood (15–24 years) [2]. Many Indigenous smokers begin their habit at a young age. In 2004–05, 10% of Australian Indigenous current and ex-smokers reported they began smoking regularly before the age of 13 years and over two-thirds before the age of 18 years [3].

Adult smoking usually has its roots in adolescence. If individuals do not take up smoking during this period it is unlikely that they ever will [4]. Moreover, once smoking becomes established, cessation is challenging; the probability of subsequently quitting being inversely proportional to the age of initiation [5]. Consequently, the prevention of the onset of adolescent smoking is a key component of efforts to reduce the overall prevalence of smoking and smoking-related morbidity and mortality. This is particularly true for Indigenous Australians for whom tobacco use is responsible for 20% of deaths [6].

Research reviews and longitudinal studies have revealed an array of often inter-related factors that are associated with smoking initiation and progression [7-10]. These include personal (e.g. age, ethnicity, substance abuse, emotional disorders, risk perceptions), family (e.g. parental smoking, parenting styles, parental attitudes towards smoking, socioeconomic status), social (e.g. peer smoking), and environmental factors (e.g. tobacco advertising, cigarette pricing). The most consistent findings in the literature relate to the influencing role of peers and family on youth smoking behaviour [11-13], while there is emerging evidence on the impact of environmental determinants such as indoor smoking bans [14,15] and social marketing campaigns [16].

Despite this wealth of literature, there is a paucity of published research that focuses on young Indigenous Australians and tobacco [17]. One qualitative study in Western Australia investigated smoking experimentation and notions of addiction among youth using focus group methods. The study included a subgroup of Australian Indigenous youth (n = 37) and found they were more likely than non-Indigenous youth to cite stress, boredom and overt encouragement from friends as reasons for smoking [18]. Overall, this study found that although adolescents had a reasonably good understanding of the concept of addiction, they did not generally regard smoking as particularly addictive at their age. An exploratory study of rural adult Aboriginal women’s experiences of smoking initiation in south-east Australia identified peer and family influences as factors contributing to smoking initiation; participants reported that smoking was normalised within extended family networks and that young women often smoked in order to be accepted among their social networks [19]. While these recently published studies have shed some light on smoking among Indigenous youth, one was retrospective and limited in its scope by gender and geographical location [19] and the other did not explore in-depth the broader social and cultural determinants of initiation and smoking [18]. There are still significant gaps in our knowledge. While there is more research in the international literature that reports on smoking uptake in other Indigenous and minority groups [20-22], they remain a relatively small proportion of the evidence base considering the burden of smoking in these specific populations. Further research is required to understand young Indigenous people’s experiences, behaviours, interactions and social contexts as they relate to smoking, especially in Australia.

The aim of this research project was to explore the determinants of smoking among Australian Indigenous young people with a particular emphasis on the social and cultural processes that underlie tobacco use patterns among this group. Specifically, we sought to understand the factors that predispose Indigenous youth to start smoking, or protect them from taking up this behaviour.

## Methods

### Research design

This project was undertaken in northern Australia between June and December 2011. It involved one urban (Darwin) and one remote site (a small mainland community of approximately 1000 residents 580 km east of Darwin). This project took a participatory approach to give young Indigenous people, an often marginalised group, both agency and a voice in research which has direct relevance for them and which may ultimately impact upon them [23].

We collaborated with a team of four trained Aboriginal ‘peer researchers’ (a male and a female in each site). An opportunity arose early in the research to involve two non-Indigenous peer researchers and recruit a smaller non-Indigenous sample. We included this sample to explore any significant differences in determinants of early smoking experiences and to elicit more data about the wider social and environmental context in which young Indigenous people start smoking. The focus on Indigenous smoking remained unchanged.

We undertook focus group discussions (FGDs) and semi-structured individual interviews. In the focus group discussions we aimed to generate a range and diversity of views on smoking initiation and to explore differing perspectives [24]. In the interviews we explored individual experiences in more depth to understand the smoking or non-smoking trajectory of individual participants [25]. Alongside these traditional qualitative methods, we also used visual methods (photography) to explore the social context and social influences of youth smoking.

In recent years, the use of visual methodologies has gained increasing prominence in social research, especially with marginalised communities. The most well known of these methodologies is ‘photovoice’ [26,27], a methodology that uses photography to promote community engagement on health and social issues. The use of photography in this project, while informed by the principles of photovoice, was employed as an individual exercise to promote reflection about the social context and social impact of smoking, as seen through the eyes of young people [28]. The photos acted as prompts for discussion about smoking and as such the method is more in line with the technique of photo-elicitation, where the emphasis is on the images as a means to unearth rich verbal data in individual interviews, rather than focusing on the visual content of the photos themselves [29].

We gave disposable cameras to 11 young people (both smokers and non-smokers) and asked them to take photos of how they experienced smoking in their everyday lives. The team then asked participants to talk about the content of their photos and their interpretations of the visual data they had created [30].

### Data collection and analysis

The young peer researchers contributed to defining the final research questions and research methods, recruited participants, undertook the data collection with the support of the research team, and assisted in interpreting the data. All peer researchers attended a two-day training workshop.

We utilised a mix of network and purposive sampling to recruit youth (13–20 years) across key sociodemographic factors: age, gender, and geographical location (urban/remote). We aimed for a mix of never smokers, experimental smokers and regular smokers in the final sample. Our primary points of recruitment were three participating schools (two in Darwin and one in the remote community). However, to include young people who might not be attending school, we recruited through the local social networks of the peer researchers and through a not-for-profit local youth centre in Darwin that caters to at-risk, mostly Indigenous youth. Youth were recruited to take part in focus group discussions initially and from this group a subset were selected for in-depth interviews based on their interest and enthusiasm for the project and ensuring a mix of ages, gender and smoking status.

While we intended to divide FGDs by gender, in most instances this was not possible because of the challenges of getting young people to commit to set times when they had many competing priorities. We ran 15 group interviews; seven of these were run as FGDs. The remaining 8 included only 2 or 3 participants owing to unforeseen circumstances for young participants at the time. In these 8 we loosely followed the focus group interview guide but commonly deviated to a deeper exploration of the personal experiences of one or more participants. We also conducted 11 photo-interviews with individual participants (in one session three Indigenous participants felt more comfortable meeting together). Towards the end of data collection, our sample included a diverse range of participants and no new themes were emerging. While we would have liked to interview more non-Indigenous smokers to compare to our Indigenous cohort, time and resources for the project did not allow this.

We pilot tested the focus group and individual interview guide with our youth researchers. Because they were all members of the eligible target group for this project, we included their interviews as key informant data. Group and individual interviews ranged in duration from 30 to 90 minutes and were held at schools, a youth centre and our research institution. Participants were reimbursed with a AUD$30 gift voucher in recognition for their time and effort. The interviews were audio-recorded and transcribed verbatim for analysis.

We used the theory of triadic influence (TTI) [31], an ecological model of health behaviour, as an organising theory for data collection and analysis. The TTI asserts that all behaviours are influenced by an interaction of genetic (nature) and environmental (nurture) factors. It divides these factors into three streams of influence on behaviour: environmental (community characteristics, media influences, legislation and policy), social (including parent and peer influences and their attitudes, use of tobacco and characteristics of relationships) and personal (genetic, biological, personality variables, gender, ethnicity and age) [8]. All three streams flow from causes more distant from the behaviour, over which individuals may not have much control, through to predictors closest (proximal) to the behaviour, providing a cascade of multiple and interacting influences. Proximal predictors are conceptualised as those that predict behaviour, while distal influences help explain it [32]. We structured the questions relating to why youth smoke in our interview guides around this framework and predictors of youth smoking found in the literature. The topics covered in our semi-structured group and individual interview guides were: age of initiation, where youth smoke and with whom, where they access tobacco at different ages and stages of smoking, why they start smoking and for regular smokers, why they continue to smoke. The individual interviews probed more deeply into individuals’ smoking or non-smoking ‘careers’ to date.

Our first level of analysis organised ‘chunks’ of textual data into open codes that arose inductively from the data. Two authors (VJ and DW) each independently coded a sub-set of the group and individual interviews and then compared coding. Code terms were discussed and refined and after a second level of analysis of the same sub-set of data, codes were grouped into categories and a category codebook was constructed. Consensus was reached through discussion and iterative review of codes and categories. This involved a process of constant comparison between and within categories as we proceeded to work through the data. A third author was available to consult if a consensus could not be reached about the categories; however, this was not needed. The first author completed the remainder of the data analysis using the codebook. The final level of analysis involved elucidating the key themes arising from the data as they corresponded to the theory of triadic influence. After this the first author discussed the findings and her interpretations with the research team, including one Indigenous peer researcher, which elicited further discussion and refinement. The content of photos was not specifically analysed in this study; instead the dialogue generated by the photos were analysed thematically as described above. Data were organised and managed using NVivo 9.

Ethical approval was given by the Human Research Ethics Committee at Menzies School of Health Research, including its Aboriginal sub-committee.

## Results

In total we interviewed 65 young people aged 13–20 years in this project. The majority (71%) were Indigenous (see Table 1 for participant demographics). Twenty-six (40%) were female. Of the Indigenous participants, 21 (46%) were smokers (inclusive of occasional and regular smokers). Of the 19 non-Indigenous participants, 3 were smokers. The majority of Indigenous and non-Indigenous participants had experimented with smoking to varying degrees. We were only able to recruit nine participants living in the remote community due to a change in staff roles at the remote community school and the involvement of peer researchers in cultural ceremony business, which meant they were unavailable for long stretches of time. However, we did recruit 22 youth who attended boarding school in Darwin but who resided in a remote community. Approximately 50% of the final sample nominated a remote community as their home. All participants attended school or were employed at the time of the study.

**Table 1:** Characteristics of youth study participants

**Characteristic**		**Study participants (n = 65)**
Age (years; mean)		15.6
Ethnicity n (%)		
	Indigenous	46 (71)
	Non-Indigenous	19 (29)
Gender n (%)		
	Male	39 (60)
	Female	26 (40)
Current smoking status n (%)		
	Smokes daily or weekly	20 (31)
	Smokes less than weekly	4 (6)
	Non-smoker	40 (62)
	Unknown	1 (1)
Home Community n (%)		
	Remote community	31 (48)
	Darwin	34 (52)

Because our primary aim was to explore the determinants of smoking initiation among Indigenous young people we focus our findings on the prominent themes that emerged from the Indigenous data and draw attention to where there are significant differences with non-Indigenous youth within these themes. Unless indicated, all quotations used to support the emergent themes came from Indigenous participants. In this study, we use the terms youth, young people and adolescents interchangeably to describe our study group (aged 13–20 years).

The findings highlight the particular role of young people’s immediate social context on smoking uptake. While there were other factors that were perceived to influence youth initiation and/or smoking progression, such as personality, stress and nicotine dependence, these were lesser themes and are not detailed in this paper.

### Starting to smoke

Participants identified different stages of smoking from first puff and experimentation, through to social or ‘casual’ smoking, and established smoking. These classifications implicitly acknowledge that starting to smoke is not a ‘one off’ event. Instead, it is a dynamic process with several stages between pre-contemplation to established (daily) smoking [4]. In this study, themes that emerged relating to smoking *initiation* (i.e. first few cigarettes) highlight the particular role of family influences. Facilitating access to tobacco, role modelling and smoking socialisation were all factors that contributed to early smoking experiences.

### ‘Trying it out’

Acquiring tobacco from family members was a common route of access for early smoking experimentation, and particularly common for the first puff, which was usually opportunistic and facilitated by the availability of tobacco in the home. Some participants were supplied tobacco directly by family members, usually older cousins or siblings. Young people also took tobacco from ashtrays, cigarette packets, or discarded cigarette butts.

In this study, participants reported that experimenting with smoking commenced usually between the ages of 10 and 13, but it was not unusual to take a first puff before this; as early as seven or eight years. Those who revealed that they initiated smoking earlier generally lived with other smokers and had greater exposure to the behaviour and more ready access to tobacco. A key motivation for experimenting with smoking was curiosity, particularly if there was high exposure among young peoples’ family and/or social networks.

Many Indigenous participants had their first smoking experience and experimented with relatives around their age or older, usually siblings or cousins. Overt pressure from older relatives to try smoking was reportedly not uncommon. Further, family members sometimes played a key role in providing instruction on smoking technique, as well as methods by which to mitigate the taste or physiological effects of tobacco smoke:

Q. Have you tried smoking before?

When I was ten. My sister was a smoker; I used to hang around her a lot. And one night she told me to put some smoke in my lung. So I did…I stole them when mum and dad were asleep. And she told me to have a puff, so I did, but then I started coughing and I said “Yuck, how do you do that?” and she said “If you keep doing it, you get used to it.” And yeah I tried, and she told me “If you swallow it and have a feed, it’s better” and yeah, so I did that…

(Female, smoker, 15 years)

The first puff was universally characterised as a ‘bad’ experience, described as “disgusting” and “yuck.” For some it was such a negative experience that they delayed trying again for a significant period of time. However, if subsequent “tries” were supported by family or peers, the negative physiological effects could be overshadowed by positive reinforcement [22]. For a few participants, the first puff was instrumental in establishing themselves as non-smokers. Generally, those for whom the physiological effects contributed to their decision not to smoke also received reinforcing messages from their family and/or friends not to smoke.

### Family as ‘teachers’

Smoking in the household and among extended family networks was prevalent for youth smokers in this study and a key theme was that of learning to be a smoker through family exposure. “Teaching [smoking] from parents” took various forms. These included being exposed to tobacco and smoking paraphernalia from an early age when asked to roll cigarettes or light a ‘smoke’ for older family members. Direct mimicry and copying adult smoking behaviour using rolled up paper or twigs was also learned through observation. There was the implicit assumption that if parents smoked, it must be sanctioned:

Oh well, as a little kid, like mucking around you know, you copy your parents, you don’t know what they’re doing, you think it’s cool, and then you’re probably like six years old and you just think how cool, I’m going to try it too.

(Female, smoker, 15 years)

Because you learn a lot when you’re growing up through visual and seeing how everything works really. So it’s accepted and the fact that your family is doing it, so yeah, must be okay if mum’s doing it.

(Male, smoker, 19 years)

This echoes previous qualitative research with Native American youth, who perceived that because smoking was so prevalent among families; it was regarded as ‘normal’ and acceptable behaviour [22] Further, a permissive or ambivalent attitude by parents of their smoking, lack of or ineffective consequences for youth smoking, and a general lack of monitoring were themes reported by smokers:

Q. Are there rules around where you smoke at home?

Yeah, just not inside, that’s basically it. And I think my brother, because he’s under 18, Mum’s doing the same thing that she did with me. [She says] “If you want to smoke, smoke outside the gate.” So yeah, I think he smokes outside the gate usually when she’s at home, but when she’s not there, he’ll go out the back with everyone else.

(Male, smoker, 19 years)

Young people reported that parents did not generally give their children tobacco or actively support their smoking behaviour. However, it was perceived that by the time young people reached their mid-late teenage years, parents often thought their children were ‘old enough’ to make their own decisions or that they were beyond parental control to influence their lifestyle choices. This scenario was more commonly described among Indigenous compared with non-Indigenous participants:

Q. Does your father know that you smoke?

I’m pretty sure he’s aware that I smoke; like my step mum does know. Every now and then when I’m stressed out because of him, I will like have one out the back or whatever. And she doesn’t care like, because my older sister does it, and she’s whatever, like she can’t stop us, we’re like older, we’re ourselves now, we’re not little kids.

(Female smoker, 15 years)

Another way in which family facilitated youth smoking behaviour was through smoking together. Sharing of cigarettes or sharing in the act of smoking has previously been found to nurture a sense of belonging and social cohesiveness among Aboriginal families and communities [33]. Similarly, in this study, some young people reported that sharing a smoke with relatives provided opportunities for socialising, ‘hanging out’ and gaining support, which also reinforced the behaviour:

So it was always good to go talk to my Aunty, because I know that she’s been through a lot through her life, so it was good to talk to her about the issues that I had in my life at the time. And yeah, it was just good to sit down and have a smoke.

(Male, smoker, 19 years)

**Figure 1:**
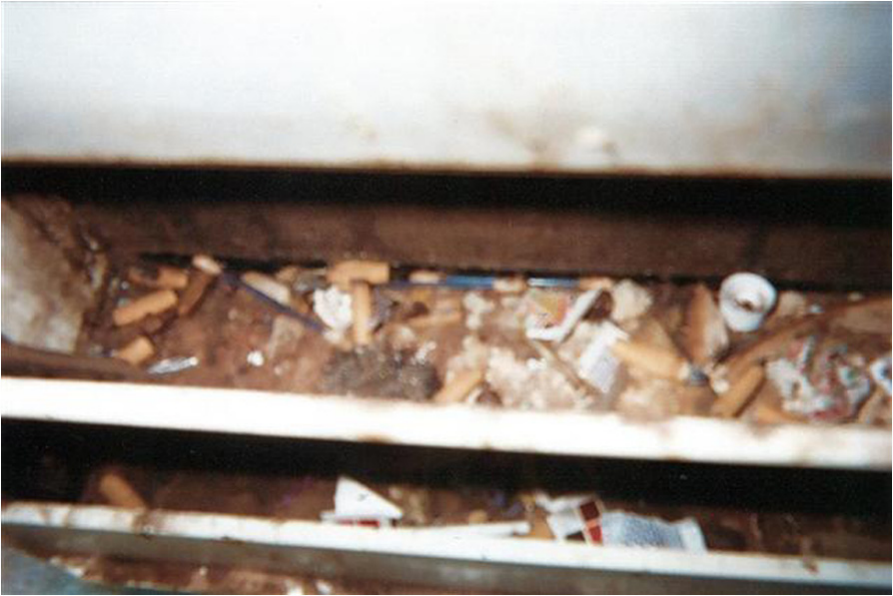
**Cigarette butts. **Three young people in the remote community site discussed this photo during a group interview. It is a photo of a window sill in a home where residents discard their cigarette butts among other rubbish. The participants reported that smoking is common in this remote community; very few households have no smokers living in them. While some households are smoke free inside, many are not.

While numbers in our sample living in a remote community at the time of the study were small, data from this group and from boarding students suggest that in the remote setting, smoking within families is normative and exposure frequent. High prevalence of smoking in remote Australia and frequent overcrowding support this [2]. Data elicited from photos taken by three remote interviewees focused on the litter from used butts and discarded cigarette packets in homes and generally around the community (see Figure 1). Smoking was also associated with other social activities in remote communities, such as gambling, where young people were provided the opportunity to win disposable income that could be used to purchase tobacco.

Findings for the non-Indigenous participants suggested that youth were similarly influenced to smoke by watching family, and frequently accessed tobacco covertly from household supplies. However, there was less indication that they regularly experimented with family members (they smoked mainly with peers) or were actively given tobacco by family members. While experimenting with family was commonplace among Indigenous participants, as described above, some did report that they smoked exclusively with friends and avoided smoking around family, because they were afraid that relatives would disclose their behaviour to parents.

Indigenous and non-Indigenous participants who smoked at the time of interview and who indicated that they had been exposed to family influences to smoke as children reported a progression in their smoking later in high school.

### A contrast: the influence of anti-smoking socialisation

While the data mostly focused on determinants of smoking, lack of access to tobacco and role modelling in the home, as well as anti-smoking socialisation from family appeared to be protective against starting to smoke. Explicit parental anti-smoking socialisation was a more significant theme for non-Indigenous, compared with Indigenous participants (although the majority of non-Indigenous participants were non-smokers). Nevertheless, the protective effect of anti-smoking socialisation, when it did occur, appeared to be the same across ethnic groups.

Both Indigenous and non-Indigenous non-smokers generally reported no or less exposure to smoking in their households. A lack of access and direct role modelling was, they perceived, a key determinant of their not becoming smokers, even though most did experiment to varying degrees.

My parents never smoked, so you’re just never really around it… So, none of us smoke… none of my brothers or sisters smoke.

(Female, non-smoker, 18 years)

**Figure 2:**
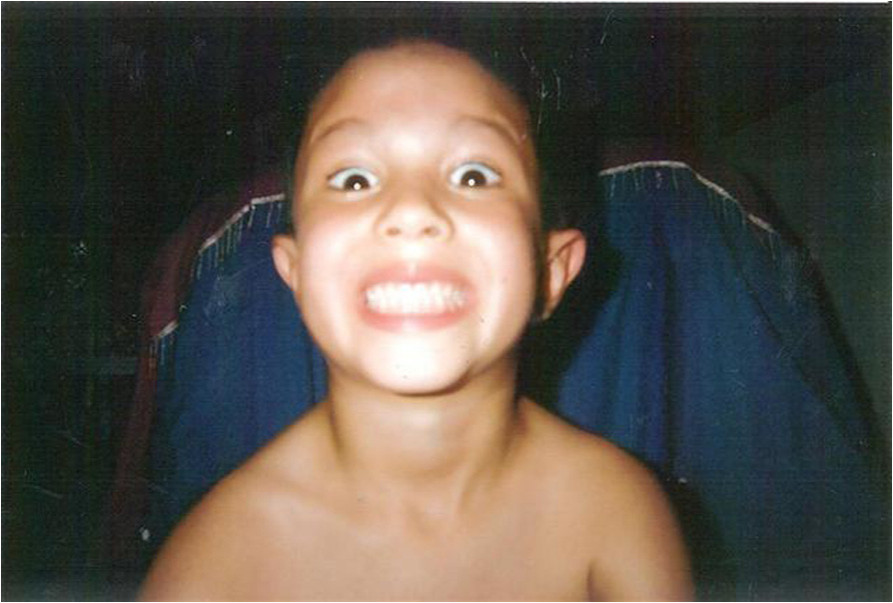
**Little brother. **My brother came along at an age where I was probably the most likely to make my mind up about smoking. I was around 11 or 12 years old and I had a lot more exposure from my friends.. but then once he came along and my mum stopped, there was just none around the house. It helped reinforce the decision not to smoke. (*Female, non-smoker, 17 years*).

Additionally, strong anti-smoking socialisation in the home was a central theme among non-smokers. Anti-smoking socialisation by parents included instituting smoke free indoor spaces, not smoking around children, strong anti-smoking messages, and clear and communicated consequences to smoking. This was true even when parents were smokers themselves and appeared to be moderated by whether youth and their parents had a positive relationship characterised by respect and trust. This theme is well illustrated by data elicited by a photo taken by Sandy (a pseudonym), one young woman in this project (see Figure 2).

Sandy is a 17 year old Indigenous young woman from a close-knit family living in Darwin. Sandy was exposed to smoking among her immediate and extended family from an early age but despite this, she was brought up not to smoke. While her mum smoked, she never did so around the children. She banned smoking inside the house and provided strong anti-smoking messages, telling them it “was a disgusting habit”. When her two younger brothers were born Sandy’s mum quit for good and this appeared to be a defining moment for Sandy. While she experimented on a few occasions, her dislike of the experience and positive family influences were reportedly central to her decision not to smoke. While many of Sandy’s aunties and uncles smoke, she reported that none of her cousins did; highlighting a generational shift among her family towards not smoking.

### Smoking as a social activity

It was during high school (approximately 13–18 years) that progression of smoking from initiation to more frequent experimentation and in some cases regular smoking was perceived to generally occur. Additionally, during this period, the influence of friends and broader social networks on smoking behaviour reportedly increased, as exposure to smoking among peers escalated and smoking assumed a fundamentally social function.

**Figure 3:**
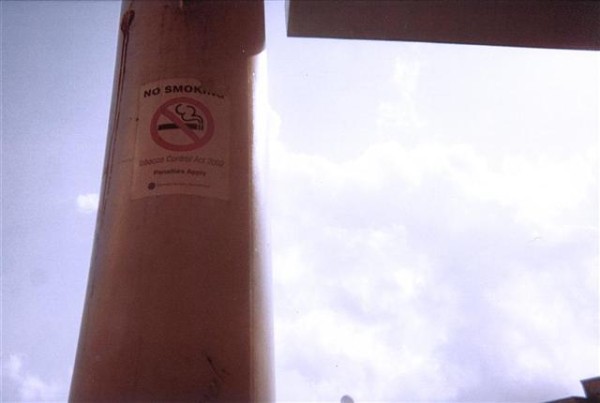
**Bus stop. **I see lots of people just having a quick smoke before they go on a bus or kids just sitting around, I don’t know, copying each other, having a smoke before and after they go to school. (*Female, smoker, 15 years*).

Smoking alone at this developmental stage was not perceived as commonplace. Instead, teenagers smoked where “everyone else smokes;” often in groups in public but out of the view of parents and teachers. In remote communities, adolescents went to secluded water holes and places in the bush to smoke. In the city they smoked at the bus stop (see Figure 3), outside the mall and the skate park - common ‘hang out’ or ‘meet up’ spots where smoking was embraced as a social activity. They also smoked at school despite universal ‘No Smoking’ policies. Participants across different schools shared stories of known secluded smoking sites behind the toilets, on the oval, in bushes on the school perimeter where smoking was commonplace.

Young people acknowledged there were greater restrictions on smokers with smoke free laws. However, the over-riding perception was that such laws did not necessarily impact on smoking initiation, especially as the smoke free regulations young people are most in contact with (at school, bus depot, outside the mall) were commonly flouted by smokers, with perceived negligible consequence. Compliance with smoke free laws in the remote context was perceived as particularly poor. Despite this, a few urban Indigenous participants did reflect on the impact of smoke free areas in denormalising smoking and impacting on behaviour. Those who perceived smoke free laws as effective in preventing youth smoking also generally reported being influenced by other anti-smoking messaging from family and/or media:

But smoking is just becoming you know, more and more banned everywhere and you just - I don’t see it that much anymore, I mean I guess that is a pretty important thing - the lack of smoking in my life is pretty significant.

(Female, non-smoker, 20 years)

As youth progressed from trying smoking for the first time to more regular smoking often during high school, avenues for acquiring tobacco broadened, as has been described elsewhere [34]. Peers became a more common means to access tobacco, although Indigenous participants in particular cited family members as a continued source of tobacco during adolescence. Friends shared smokes, went halves, and ‘bummed smokes’ off one and other; behaviour that reinforced social bonding through shared experience and consequently reinforced smoking.

Other sources of tobacco included older friends or sometimes strangers who were approached to purchase tobacco. Youth also reported the ability to access a black market where cigarettes were purchased as single sticks at an inflated price. While this practice has been previously identified in remote settings, [33] this was also reportedly a means to access tobacco for both Indigenous and non-Indigenous youth across schools in the urban setting. Additionally, it was reportedly not uncommon for under-aged youth to purchase tobacco directly at outlets; usually ‘known’ small corner shops where identification of age is rarely required: the larger retail outlets were avoided. This emphasises findings from previous research that has reported that youth are adept at finding outlets that are prepared to sell tobacco to minors [35] and the difficulties with enforcing bans on sales to underage purchasers [36].

### Starting to smoke to ‘fit in’

Participants noted that during high school years, social pressure to smoke was an increasingly influential determinant of experimentation and progression of smoking. The process of peer socialisation, whereby adolescents take on the values and behaviours of the ‘group’ in order to be accepted [37], was a theme that cut across Indigenous and non-Indigenous participants, but was a more central theme for female participants generally.

There were differing perceptions as to the prevalence of overt pressure to smoke. Nevertheless, a range of participants did report feeling ‘forced’ into smoking on one occasion or more; the consequences for not smoking included ridicule and humiliation. However, a more consistent theme was that peer socialisation worked more through indirect pressure to conform to social norms, rather than peers providing direct encouragement to smoke. Some young people smoked so as to ‘fit in’ with friends, to avoid being the ‘odd one out’ or an ‘outcast’ among peers:

“They want to be the same as the other ones who smoke…Because if you are a non-smoker and you see them over there, and they are your friends, it doesn’t suit you if you are not smoking. But if you start smoking, it’s like you are a member of that group.”

(Male, non-smoker, 20 years)

Others started to smoke to project or maintain a certain image, again generally to be accepted by a specific group or crowd, or to attract the opposite sex. Smoking in this context played a functional role in assisting young people to reflect an image that was “rebellious,” “cool”, or “grown up”:

Oh well, I grew up running around and yelling out gang names… Yeah so for me it was something to fit in with the group. Now I’m addicted and can’t get off it. So now I’m swearing because it costs me $20.00 a day.

(Female, smoker, 17 years)

“They’re growing up, they think they getting smarter and smarter, like an adult, becoming a woman and not a girl anymore.”

(Female, smoker, 20 years)

Conversely, non-smokers commonly described smoking in pejorative terms, describing it as “gross,” and “disgusting,” and this negative imagery was a key reason given for not starting to smoke. This characterisation of smoking was more dominant among non-Indigenous than Indigenous participants, perhaps reflecting the difference in the degree to which smoking is denormalised in the majority population. Nevertheless, some Indigenous participants reported similar views, especially if they had also received strong anti-smoking messages from their families:

It’s sort of switched from cigarettes being cool to cigarettes being just disgusting and really not, yeah, not cool at all…That’s how I see it.

(Female, non-smoker, 17 years)

Participants perceived that a negative image of smoking had progressively developed as a consequence of the behaviour being less common in the community than it once was. A perceived drop in prevalence, increasing restrictions on smokers as a consequence of smoke free areas, and graphic pack warnings have all assisted in denormalising and to an extent stigmatising smoking, in some instances stigmatising the smokers themselves. This had implications for not only how non-smokers perceived smoking but also how non-smokers related to smokers themselves:

My brother’s like that. If a girl smokes, he doesn’t want a bar of it. It’s just a really big turn off.

(Female, non-smoker, 18 years)

Participants also highlighted the particular role of alcohol, usually in the context of social gatherings, in facilitating smoking. Smoking in combination with marijuana was also reported, highlighting the common co-occurrence of tobacco, alcohol and cannabis use in adolescence [38]. Alcohol use promoted participation in social gatherings in which access and availability of tobacco was increased and social inhibitions and control reduced. Youth who smoked infrequently in the context of social gatherings, and often in association with alcohol were commonly defined as ‘social’ smokers, regardless of the regularity of their smoking behaviour.

### The reinforcement of social networks

Related to the theme of peer influence on smoking initiation, is the role that peer behaviour played in maintaining smoking (or non-smoking) behaviour. In the previous section, we described how adolescents reported being socialised to smoke by the influencing norms and behaviours of their social group (peer socialisation). Another avenue through which peer influence led to group homogeneity in smoking and other behaviours is ‘peer selection.’ This describes the process whereby young people gravitate towards or select social networks with similar norms and behaviour to their own [39]. This is exemplified in the following quote, where a young male smoker described how he was ‘encouraged’ to seek out other smokers, as a consequence of feeling marginalised by the wider school community. In this instance the ‘smokers’ group’ was described as a separate entity with inclusion predicated on smoking status and members exhibiting strong social bonding by virtue of being excluded from the mainstream:

In school, I mean, smoking was something that was frowned upon by most people, so I did feel singled out at that point as well as a smoker, which encouraged me more to hang around with more smokers and begin the cycle of more and more cigarettes going in to my body too …Like the whole smoking group socialised together and we all mixed in after a while because there was no point in being separated because we were all singled out anyway…

(Male, smoker, 19 years)

This participant’s social context, while providing him with a supportive environment, also contributed to a progression in smoking intensity. This is a reminder of how universal efforts to denormalise smoking may potentially cement smoking in the lives of some youth who find themselves excluded by social practices that are progressively viewed as ‘deviant’ and unacceptable [40].

**Figure 4:**
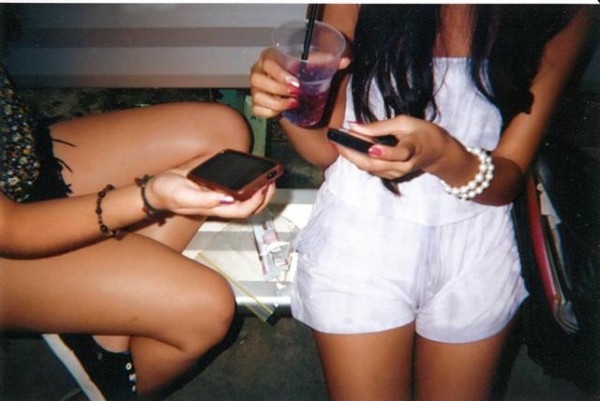
**Friends. **So this is two of my best friends. And so this is at lunch time when a lot of smokers do go for smokes as well. We find other ways to entertain ourselves. So they have their phones out, food, just talking. No need for cigarettes. And sometimes we study during lunch as well. Yeah. My friends don’t smoke, I don’t smoke. These are the people that I’m like really closely knit with. (*Female, non-smoker, non-Indigenous, 15 years*).

Socialising processes that may encourage adolescent smoking also operated to protect young people from smoking [41], as highlighted by data elicited from a photo taken by one young non-Indigenous woman interviewed for this project (see Figure 4). Talking about the image, she explained that her group of non-smoking friends entertained themselves with other activities during school breaks when smokers commonly go for a smoke. As a collective, they found no “need for cigarettes” in their lives and these distinguishing values and behaviours consistently reinforced the group as non-smoking.

Indeed, peers in non-smoking peer groups were often cited as a source of sometimes vehement anti-smoking messages and demonstrated the power of indirect pressure to conform to actual or perceived social norms, particularly in this age group. This was a lesser theme among Indigenous participants but was nevertheless present, as exemplified in the following excerpt, where a young Indigenous woman recalled the negative reaction of her friends on the few occasions she experimented with smoking at parties:

My close friends disapproved highly…they sort of thought that I got what I deserved the next day, from being sick, they weren’t really that sympathetic they were like well, “that’s what you get.” So I guess, like, I think that helped in me not smoking as well; my close friends didn’t approve of smoking at all, they thought it was trashy and they really talked it down a lot.

(Female, non-smoker, 20 years)

## Discussion

The findings of this study revealed that for Indigenous (and non-Indigenous) young people, their immediate social environment, that is, family and peer networks, played a central role in smoking initiation and progression. This highlights the social stream of influence within the TTI framework on youth smoking behaviour in this context.

Flay, Snyder and Petraitis [32] identify that within the social stream, the ‘ultimate cause’ of youth smoking is the social context in which an individual lives. Context determines the breadth, extent and nature of interpersonal interaction [42]. This flows through to and interacts with the next level of influence at the social-personal nexus, where smoking behaviour is influenced by social bonding to significant others and observed (modelled) behaviours. Family and peer groups have a key role at this level of influence, as this study’s findings demonstrate. The experiences and the information youth gain within these social networks inform and shape their understanding of what is normative and acceptable behaviour [40]; social normative beliefs about smoking subsequently contribute to young people’s decisions or intentions to smoke [32].

Our study did not yield detailed information about the broader social context in which youth start to smoke. However, our findings that high exposure to smoking role models as well as to activities that may facilitate tobacco use (e.g. gambling), coupled with perceived poor compliance of smoke free areas in the remote Indigenous context, may shape the interpretation of social norms related to smoking in different ways to urban youth. At the next level of influence, both general parenting practices and smoking-specific practices influenced the development of young peoples’ social normative beliefs around smoking and subsequent smoking behaviour. Lack of, or inconsistent consequences for smoking, was reported by smokers in this study. Similarly, previous research has found that children of parents who have an ‘unengaged’ or more permissive parenting style are more likely to smoke, compared with children whose parents have a more ‘authoritative’ style of parenting (i.e. set clear limits for behaviour, as well as monitor compliance) [43,44]. In this study, low levels of parental efficacy in reducing teen tobacco use and lenient household rules about smoking in the home was also reported, despite parents often providing contradictory anti-smoking verbal messages. Focus group and cross-sectional research with a Native American population in the US suggest that these Indigenous parents may also have more lenient anti-smoking socialisation beliefs compared with other ethnic groups [45,46]. However, this was found to vary more by education level of the parent than by ethnicity [46], suggesting that socioeconomic and not ethnic status is the more influential determinant of such beliefs.

Related to the theme of parenting, smoking-specific practices within families, including role modelling smoking, facilitating access to tobacco and socialisation into smoking were also influential in smoking uptake among youth. Modelling smoking behaviour was central to how young people ‘learnt’ to smoke, consistent with the well established research finding that parent and sibling smoking is a strong and significant predictor of the risk of smoking uptake by children and young people [47]. Family as both a direct and indirect source of tobacco was also a significant finding in our study, as has previously been reported among minority and Indigenous ethnic groups in the US [20]. Socialisation of youth to smoking by other family members included the active initiation of young people to smoking and sharing in the act of smoking. In the Indigenous context particularly, the role of older siblings and cousins in this socialisation process cannot be overlooked. They were frequently the source of tobacco and the instigator of smoking experimentation for young people in the family environment; this has also been reported in other minority and Indigenous ethnic groups [22,48]. While role modelling and access to tobacco were also influential for non-Indigenous youth, they did not report the same degree of active socialisation to smoking as did Indigenous participants.

In contrast to the above, families who engaged in anti-smoking socialisation were reportedly successful in establishing norms around non-smoking and subsequently protecting youth against smoking uptake. Henriksen and Jackson , p.87 [49] define *anti*-smoking socialisation as “the transmission of knowledge, attitudes and skills that prepare children to resist smoking”. This can take several forms: establishing household smoking bans, monitoring children’s behaviour and establishing clear expectations of negative consequences for smoking, as well as expressing anti-smoking messages [50]. In this study, young children who were raised in households with fewer smokers and/or whose family members provided strong anti-smoking socialisation generally reported less inclination to try smoking and if they did try, to progress beyond experimentation. This was particularly the case if parents were non-smokers but appeared to hold, even if parents smoked. Several robust epidemiological studies have upheld the hypothesis that anti-smoking socialisation is protective against youth smoking [50-52]. Further, in this study the effect of these parenting practices appeared to be influenced by the strength of family ties, suggesting an interaction between general and smoking-specific parenting practices, and highlighting the role of social bonding in influencing normative beliefs about smoking.

The other significant influence on social norms around smoking in this study was the peer group. There is no clear consensus in the literature as to the relative importance of family and peer influence on adolescent smoking at different stages of smoking. Some reports suggest that the effect of family smoking is particularly relevant for younger children [53,54], whereas peer group behaviours are more important in influencing smoking during teenage years [55,56]. More recent longitudinal research suggests parental influences are important for initiation *and* escalation of smoking [57,58]. Peer behaviour too, has been found to affect initiation, progression and trajectories [42].

Our qualitative design was not able to ‘unpack’ the relative contribution of family and peers on smoking at different stages in this context. However, the data suggest that family influences were particularly salient for smoking initiation and experimentation but also appeared to set the foundation for some youth to progress to more regular smoking during their teenage years, or conversely not to continue beyond experimentation. Peers appeared more influential during adolescence, a critical time of transition to physical and emotional maturity and to a coherent sense of self [59].

We found evidence for both peer socialisation and peer selection and both significantly influenced social norms around smoking. These processes not only affected smoking initiation but also continued to reinforce smoking beyond initiation. Similar to the two earlier qualitative studies that included Australian Indigenous youth [18,19], we found that peer socialisation is more a normative process and less one of overt pressure to smoke [42]. Smoking to ‘fit in’ with peers highlights that group membership in adolescence confers significant benefits of acceptance and friendship, but can also require conformity in both attitudes and behaviours, which may be detrimental to health [60]. A related theme is the role that smoking plays in the creation or experimentation of different social identities [61,62] during this developmental stage. In this study, smoking was used by Indigenous and non-Indigenous participants to reflect a range of social identities from rebelliousness to ‘grown up’; identities that conferred symbolic capital within their various social contexts [40]. While smoking was used as a ‘style tool’ by some youth to communicate identity and status, it was regarded by others as a “stigmatising liability”, p.77 [40], influencing normative beliefs *against* smoking. This finding was more pronounced among non-Indigenous participants.

Our study also found that there is substantial peer group homogeneity in respect to adolescent smoking [63] with smokers and non-smokers separately ‘clustering’ [42] in close friendship networks. Peer group membership reinforced social norms around smoking behaviour, acting to reinforce or protect against smoking depending on the composition of the group. This further emphasises that smoking, contrary to being an ‘individual’ lifestyle choice, is instead enmeshed in collective patterns of consumption, and selected from among what is “socially feasible” so as to construct and maintain a social identify that expresses difference both among and between social groups, p.61 [64]. What this study also highlights is that in a context of falling smoking prevalence, peer influence can also be protective [65]. This was particularly the case for non-Indigenous participants who were non-smokers but there is evidence of changing social norms among Indigenous youth as well.

There are limitations to this study. We only included a relatively small sample of non-Indigenous participants, and within this sub-group we were only able to recruit a small number of smokers. This means that we were not able to provide a more nuanced comparison across ethnic groups but instead have focused our analysis on the major themes arising for Indigenous youth and the significant similarities and differences between the two ethnic groups. We found few marked differences in the perceptions and reported experiences of smoking by gender, although female participants appeared to be more strongly influenced by peer smoking than boys [42]. However, if we had conducted separate group interviews for females and males as planned, we may have uncovered more subtle gender differences in smoking behaviours, as has been reported elsewhere in the literature [66]. Additionally, our findings are more representative of the perspectives of youth in school or employment, which restricted our ability to explore in-depth differences across socioeconomic status, and therefore limit the generalisability of the findings. Finally, the qualitative nature of the study means we must caution against inferring causality between suggested determinants and smoking behaviour of participating youth. Social desirability may have biased participants’ responses and led them to self-censor their actual views. In addition, participants were volunteers who may have different smoking-related attitudes and experiences than Indigenous and non-Indigenous youth in the community.

Despite the limitations, this study is one of the first in Australia to provide in-depth data on the qualitative determinants of smoking among contemporary Indigenous young people. We found that family and peer social influences are particularly salient in smoking uptake among Indigenous youth, emphasising the importance of the social stream of influence within the TTI in this context. Our findings also suggest that the types of social influences to smoke were similar between Indigenous and non-Indigenous youth but that these influences were more pervasive (especially in the family domain) among Indigenous youth. This reflects the fact that Indigenous smoking prevalence is double non-Indigenous prevalence and smoking in many Indigenous families and communities remains a normative social practice [19,33]. The conclusion we draw is that higher rates of smoking uptake among Indigenous Australians are likely attributable to known causes of smoking initiation [67].

## Conclusions

Our findings have implications for both future research and practice. One important avenue for research is to explore the range of responses and beliefs regarding youth smoking from the perspective of Indigenous parents of children and adolescents, as they were excluded from our recent study and we relied solely on young peoples’ reporting of these. This is important given the role of general parenting and smoking-specific practices on youth smoking uptake. Longitudinal research with Indigenous youth to explore both the generalisability of these findings and the differential contribution of family and peer influences on smoking at different stages would be valuable; this may have implications for preventative interventions at different stages of smoking.

Future smoking prevention activities need to focus on changing social normative beliefs around smoking, both at a population level (through smoke free policies and laws and social marketing campaigns) and within young peoples’ immediate social environment. Such activities would complement other effective initiatives to prevent youth smoking, such as increasing the price of cigarettes [68]. Currently, all Australian states and territories have banned smoking in enclosed public places, particularly workplaces and restaurants [69]. The Northern Territory has traditionally lagged behind other jurisdictions in implementing smoke free areas. For example, if a majority of staff at a NT school campus agree, the school can designate a discrete outdoor area for smoking if it is not in the line of sight of children. This is in contrast to all other states and territories in Australia that ban smoking on all government school grounds by Education Department policy. The NT Department of Education and Children's Services should consider following other jurisdictions in making the whole of school campuses smoke free. The NT Tobacco Control Regulations should also be amended to remove this exemption relating to NT schools. Importantly, schools should not only implement but enforce smoke free policies, as enforcement of policy (not its existence) is necessary for it to be effective in reducing smoking prevalence among youth [1].

Another avenue through which schools might intervene to reduce youth smoking is to further explore interventions designed to alter social norms within established peer groups and harness the power of positive peer influences to reduce youth smoking. This has been successfully trialled in the United Kingdom. Drawing on ‘diffusion of innovation’ theory, the Stop Smoking in Schools Trial (ASSIST) utilised trained influential school students to act as positive peer supporters during informal (out of classroom) interactions to encourage young people not to smoke [70]. The study found a 22% reduction in the odds of being a regular smoker in intervention, compared to control schools for two years after its delivery [71].

Another obvious area for attention is the family unit, where interventions could be targeted to encourage positive parenting practices, both generally, as well as smoking-specific practices [42]. A Cochrane review of the effectiveness of interventions to help family members strengthen non-smoking attitudes and promote non-smoking by children or adolescents, found that while the evidence base is limited, some well-executed RCTs show family interventions may prevent adolescent smoking [72]. Health practitioners in contact with Indigenous families should be promoting smoke free homes and other anti-smoking socialisation behaviours.

In conclusion, it is encouraging that this study provides some evidence for changing social norms relating to smoking among young Indigenous Australians. Measures to continue to denormalise smoking and to support families to socialise their children against smoking youth should contribute to reducing smoking uptake in this population and make significant inroads into reducing the disease and death caused by smoking in Indigenous communities.

## Competing interests

The authors declare they have no competing interests.

## Authors’ contributions

All of the authors made contributions to the conception and design of the ‘Starting to Smoke’ study. VJ, DW and CE were responsible for data collection and analysis and all authors contributed to the interpretation of the findings. VJ drafted the manuscript; the remaining authors critically reviewed it and made revisions. All authors have approved the final manuscript.

## Pre-publication history

The pre-publication history for this paper can be accessed here:

http://www.biomedcentral.com/1471-2458/12/963/prepub
